# Cross-species transfer of sensor and helper NLR confers resistance to black rot in *Brassica oleracea*

**DOI:** 10.1186/s43897-026-00235-w

**Published:** 2026-07-03

**Authors:** Siping Deng, Xiaofei Du, Hongxue Ma, Limei Yang, Mu Zhuang, Yong Wang, Jialei Ji, Yangyong Zhang, Hailong Guo, Honghao Lv

**Affiliations:** 1 State Key Laboratory of Vegetable Biobreeding, Institute of Vegetables and Flowers, Chinese Academy of Agricultural Sciences, Beijing, 100081 China; 2 Department of Plant Pathology, China Agricultural University, Beijing, 100081 China

Plant immune systems utilize immune receptors to detect pathogens and initiate defense responses (Jones and Dangl 2006). These receptors can be broadly categorized into cell-surface localized pattern recognition receptors (PRRs) and intracellular immune receptors (NLRs), each playing a distinct role in microbe/pathogen-associated molecular patterns (M/PAMPs) and effector recognition, respectively (Boutrot and Zipfel 2017; Han 2019). Plant immune receptors are major breeding targets for improving disease resistance in crops. Breeding for disease resistance often involves introducing novel immune receptor genes, mostly NLRs, into crop varieties to enhance their ability to recognize and combat specific pathogens (Rodriguez-Moreno et al. 2017).

An emerging view in plant immunity highlights the concept that functionally specialized sensor and partner/helper NLRs combine together against diverse plant pathogens. Sensor NLRs detect pathogens effectors, while partner/helper NLRs mediate downstream immune signaling (Feehan et al. 2020). Sensor NLRs from one family mostly fail to function in distantly related species, a phenomenon called restricted taxonomica functionality (RTF) (Tai et al. 1999). One major reason for that is the due to the absence of necessary partner/helper NLRs. However, a recent study successfully enable rice to achieve resistance against *Xanthomonas oryzae* pv. *oryzicola* (*Xoc*) by cross-species co-transfer of *Solanaceous* sensor NLR Bs2 and helper NLR NRC (NLR required for cell death) genes (Du et al. 2025). Such approach holds great promise for revolutionizing disease resistance engineering across diverse crops in the future.

The black rot disease caused by *X. campestris* pv. *campestris* (*Xcc*) severely threatens global productions of cruciferous crops, particularly *Brassicas*. Natural resistance is very rare in these species, which greatly hinders the breeding process (Kong et al. 2021). The *Solanaceous* sensor NLR Bs2 recognizes AvrBs2 from *X. campestris* pv. *vesicatoria* (*Xcv*) and requires downstream helper NLR NRCs to activate effector-triggered immunity (ETI), which has been well characterized (Wu et al. 2017). However, whether this system can be employed in the resistance re-construction of *B. oleracea* to *Xcc* is unknown. Here, we used this approach to address the black rot disease in *B. oleracea*.

We first investigated the functional role of AvrBs2 in *Xcc* mediated *B. oleracea* infection by generating an AvrBs2 knockout mutant (*ΔAvrBs2*) from the wild type *Xcc* strain JY (Figure S1a). Using needle inoculation, we challenged three *B. oleracea* lines with contrasting resistance levels (highly resistant MY, moderately resistant 87–534, and highly susceptible CB-516) with both strains. Comparative analysis revealed significantly reduced lesion area ratio and bacterial biomass in plants inoculated with *ΔAvrBs2* compared to JY across all varieties, demonstrating that AvrBs2 deletion substantially attenuates *Xcc* pathogenicity (Fig. 1a-c). In contrast to the avirulence protein’ role in *Xcv* infections of pepper (Medina et al*. *2018), AvrBs2 functions as a virulence protein in *Xcc* infection of *B. oleracea*. This is due to the absence of NLR genes in *B. oleracea* that can recognize AvrBs2 and trigger ETI.

**Fig. 1:**
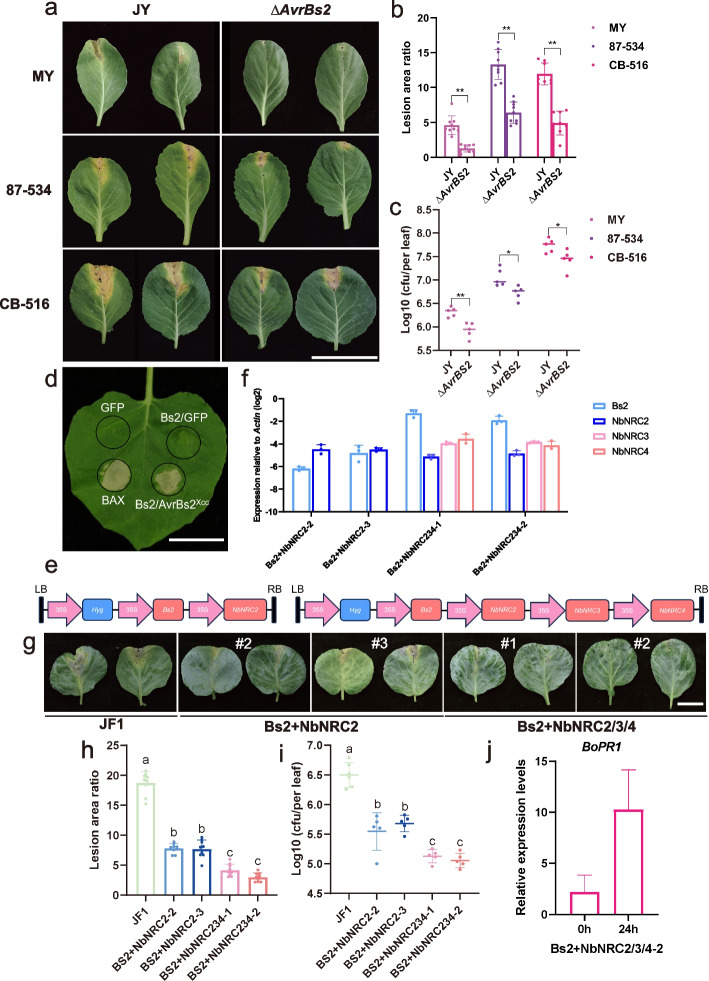
Co-expression of sensor + helper greatly enhances black rot resistance in *B. oleracea*. **a** Representative disease symptoms in *B. oleracea* leaves, inoculation with JY or *ΔAvrBs2*. Images of lesion expansion were taken at 10 dpi. Scale bar, 5 cm. **b** Quantification of lesion area ratio caused by *Xcc* in MY, 87–534 and CB-516. Lesion -area ratio was measured at 10 dpi. Individual data points are shown with mean ± SD (*n* = 9). **c** Bacterial growth of JY and *ΔAvrBs2* in MY, 87–534 and CB-516. Bacterial populations were determined at 4 dpi. Individual data points are shown with mean ± SD (*n* = 5). **d** Cell death induction by co expressing Bs2 with AvrBs2^*Xcc*^. Scale bar, 3 cm. **e** Vector architecture of the constructs: p35S:Bs2 + p35S:NRC2 and p35S:Bs2 + p35S:NRC2/3/4. **f** RT-qPCR analysis of transgenic *B. oleracea* lines. **g** Representative disease symptoms after *Xcc* inoculation. Leaves of JF1 and transgenic lines were inoculation with strain JY. Images of lesion expansion were taken at 10 dpi. Scale bar, 5 cm. **h** Quantification of lesion area ratio caused by *Xcc* in JF1 and transgenic lines. Lesion area ratio measured at 10 dpi. Individual data points are shown with mean ± SD (*n* = 9). **i** Bacterial growth of *Xcc* in JF1 and transgenic lines after inoculation. Bacterial populations were determined at 4 dpi. Individual data points are shown with mean ± SD (*n* = 5). **j** Relative expression levels of the *BoPR1* upon *Xcc* inoculation

The AvrBs2 is highly conserved in sequence and function among *Xanthomonas* species (Li et al. 2015). Notably, the AvrBs2 protein in *Xcc* exhibits high sequence similarity to that in *Xcv* (Figure S1b). To investigate whether Bs2 can recognize AvrBs2^*Xcc*^ in a similar to AvrBs2^*Xcv*^, we conducted a transient expression assay in *Nicotiana benthamiana*. The results showed that while the expression of Bs2 alone produced no phenotypic response, co-expression of Bs2 with AvrBs2^*Xcc*^ triggered a clear cell death response (Fig. 1d). This demonstrates that AvrBs2^*Xcc*^ is also recognized by the Bs2.

NRC helper NLRs are only found in asterids and absent in *Brassicaceae* family. To confer black rot resistance in *B. oleracea* by introducing *Solanaceae* NLR genes, we then generated two sets of expression vectors using the Golden Gate assembly method: (1) p35S:Bs2 + p35S:NbNRC2, and (2) p35S:Bs2 + p35S:NbNRC2/3/4 (Fig. 1e). These constructs were transformed into JF1 via *Agrobacterium*-mediated transformation, with transgenic lines identified through RT-qPCR (Fig. 1f). For pathogen resistance evaluation, we needle inoculated transgenic lines and JF1 controls with *Xcc* strain JY. JF1 developed expanding brown necrotic lesions, while Bs2 + NbNRC2 lines showed significantly reduced area ratio and bacterial biomass, demonstrating partial resistance. Strikingly, lines expressing Bs2 with NbNRC2/3/4 exhibited minimal white transparent lesions without spreading, along with dramatically reduced biomass, indicating a robust ETI response (Fig. 1g-i). Consistent with an activated immune response, the expression of the defense-related marker gene *BoPR1* was significantly elevated in inoculated Bs2 + NbNRC2/3/4 lines (Fig. 1j). Contreras et al. (2023) demonstrated that the sensor NLR Bs2 triggers oligomerization of the helper NLR NbNRC2 upon recognition of AvrBs2. Notably, the observed NbNRC2 oligomers did not correspond to integer multiples of the monomeric protein size, implying potential incorporation of additional protein components within the NbNRC2 immune complex. Du et al. (2025) also demonstrated that co-expression of Bs2 and NbNRC2 in rice confers partial resistance against *Xoc* infection, while combined expression of Bs2 with the NbNRC2/3/4 triad establishes complete immunity.

In summary, our study establishes that AvrBs2 acts as a virulence protein during *Xcc* infection of *B. oleracea*, and crucially, *B. oleracea* lacks endogenous NLR genes capable of recognizing AvrBs2 to initiate ETI. To address this limitation, we successfully introduced the *Solanaceae* sensor NLR Bs2 and helper NLRs NbNRC2/3/4 into *B. oleracea*, which confers complete resistance. These findings not only demonstrate the successful engineering of black rot resistant *B. oleracea* lines but also significantly expand the cross-species applications of sensor and helper NLRs strategies in crop improvement.

## Supplementary Information

Supplementary Material: Experimental Procedures and Supplementary Fig. 1.

## Data Availability

The datasets used during this study current study are available from the corresponding author on reasonable request.
