# Supplementary material for: Cross-species transfer of sensor and helper NLR confers resistance to black rot in Brassica oleracea
Source: Mol Hortic. 2026 Jul 3;6:49. doi: 10.1186/s43897-026-00235-w (PMC13330410; doi:10.1186/s43897-026-00235-w)
Supplement: Supplementary file 1 — Supplementary Material: Experimental Procedures and Supplementary Fig. 1. [file 43897_2026_235_MOESM1_ESM.docx]

**Construction of ΔAvrBs2**

The AvrBs2 flanking fragment was amplified from the JY strain using PCR and subsequently ligated into the PK18 plasmid to create a recombinant plasmid, which was then introduced into the JY strain via conjugation with parental strains. The specific steps are detailed below: 1. The recombinant plasmid was transformed into competent *Escherichia coli* strain S17. After selecting single colonies and validating them by PCR, this strain was designated as the donor, while the rifampicin-resistant JY strain served as the recipient. 2. The donor and recipient strains were cultured separately in liquid medium supplemented with kanamycin and rifampicin until an OD600 of 0.6-0.8 was achieved. 3. 2 mL of culture from each strain were collected and centrifuged at 4000 rpm for 3 minutes, after which the supernatant was discarded. 4. The cell pellet was resuspended in sterile double-distilled water (ddH2O), centrifuged again at 4000 rpm for 3 minutes, and the supernatant was discarded. This step was repeated one more time. 5. The cell pellet was resuspended in 800 µl of sterile ddH_2_O. 6. The strains were mixed at a donor-to-recipient ratio of 1:3, and 100 µl of the mixed culture was spread onto NAN (no sucrose NA) medium and incubated for 2 days. 7. The plates were retrieved, and 1 mL of sterile ddH_2_O was added. Cells were scraped off using a spreader and then resuspended. 8. The bacterial culture was diluted in three increments: 10-fold, 100-fold, and 1000-fold. 9. A volume of 100 µl was taken from each dilution increment and spread onto NAN+Kan+Rif plates, then incubated at 28 °C for 3 to 4 d. 10. Single colonies were selected and streaked onto NAN+Rif+Kan and 10% sucrose NA+Rif+Kan plates, incubated at 28 °C for 1 day. Strains that grew only under the former conditions and not the latter were selected. 11. The selected strains were cultured overnight in NBN. The culture was then diluted 10-fold, 100-fold, and 1000-fold before being spread onto 10% sucrose NA+Rif+Kan plates, which were incubated at 28 °C for 3 d. 12. Single colony PCR validation was conducted. Clones that failed to amplify AvrBs2 were classified as positive.

**Construction of Vectors for Genetic Transformation of *Brassica oleracea***

Vectors were constructed using the Golden Gate method. Initially, the genes Bs2, NRC2, NRC3, and NRC4 were assembled into the level 0 vector. Subsequently, these genes were transferred from the level 0 vector to the level 1 vector, which contained the p35S promoter and various terminators. The expression frames containing Bs2 and NRC2, along with a dummy sequence, were then transferred to the level 2 vector. This resulted in the creation of the p35S:Bs2+p35S:NRC2 vector, which is suitable for genetic transformation of *B. oleracea*. Furthermore, the expression frames of Bs2 and NRC2/3/4 were also transferred to the level 2 vector, yielding the p35S:Bs2+p35S:NRC2/3/4 construct.

**Transgenic transformation of *B. oleracea***

Select medium-sized, plump, and sterile seeds, and place them into sterile 50 mL centrifuge tubes. Wash the seeds with 75% ethanol for 3 min, followed by treatment with 8% NaClO for 7 min. Finally, rinse the seeds three times with sterile water for 3 min each time. Allow the seeds to dry on sterile filter paper before evenly spreading them onto the germination medium (4.43 g/L MS medium, 28 g/L sucrose, and 8 g/L agar; pH = 5.8). Seal the tubes and place them in a light incubation chamber with a photoperiod of 16 hours of light and 8 hours of darkness for 5 to 7 d. Use a scalpel to cut hypocotyl segments approximately 1 cm in length and lay them flat on the pre-culture medium, which consists of germination medium supplemented with 1 mg/L 6-BA and 0.1 mg/L NAA. Incubate for 2 d. Prepare the Agrobacterium infection solution by activating Agrobacterium containing the recombinant plasmid. Mix 200-300 µL of the bacterial suspension with 50 mL of liquid culture medium, which contains 50 mg/L Kanamycin and 100 mg/L Rifampicin, in a sterile conical flask. Incubate at 28°C and 2000 rpm until the optical density (OD600) reaches 0.4-0.6. Centrifuge the bacterial suspension twice. After the final centrifugation, add liquid MS medium containing 100 µmol/L acetosyringone to infect the explants for 10 minutes, ensuring continuous shaking. Next, lay the explants flat on sterile filter paper to dry. Subsequently, place them in the co-cultivation medium, which is pre-culture medium supplemented with 100 mmol/L acetosyringone, and incubate in the dark for 48 h. Transfer the explants to the delay medium, which consists of pre-culture medium supplemented with 300 mg/L timentin, and maintain normal culture conditions for 5-7 d. Subsequently, transfer all explants to the selection medium, consisting of pre-culture medium supplemented with 300 mg/L timentin and 5 mg/L hygromycin. Perform selection over three cycles, each lasting 14 d. Harvest the resistant shoots that have developed on the selection medium and transfer them to the elongation medium, which is germination medium supplemented with 0.2 mg/L 6-BA, 0.1 mg/L NAA, and 300 mg/L timentin. Finally, transfer the shoots to the rooting medium, comprising germination medium enriched with 0.1 mg/L NAA, 0.1 mg/L IBA, and 300 mg/L timentin.

**RT-qPCR**

RNA was extracted from the leaves of transformed plants using an RNA extraction kit (Tiangen, Beijing, China). Reverse transcription was performed using a reverse transcription kit (Vazyme, Nanjing, China), with the procedure strictly following the instructions provided. The obtained cDNA was diluted to 300 ng/μl with sterile ddH_2_O for qPCR.

**Plant materials and growth conditions**

All inoculation materials were sown in plug trays and grown in a solar greenhouse, where they received standard management, and watered every 2 d.

**Inoculation test**

The black rot pathogen used for inoculation was preserved in PSB medium consisting of 10 g/L peptone, 10 g/L sucrose, and 1 g/L sodium L-glutamate, with a pH of 7.0. The pathogen was cultured at 28°C with a shaking speed of 200 rpm for approximately 12 hours, followed by dilution with sterile water to achieve an OD600 of 0.2. Once the seedlings developed 4 to 6 true leaves, a sterilized toothpick was dipped into the bacterial suspension and used to uniformly prick three times along the main leaf vein, positioned 0.5 to 2 cm from the leaf tips on three similarly sized leaves in close proximity. The inoculated seedlings were cultured at temperatures ranging from 25 to 28°C, and the incidence of disease was assessed 10 d later.

**Resistance Evaluation**

Utilize Image J v 8.0 (National Institutes of Health, Bethesda, MD, USA) to quantify the area of lesions and the total leaf area, subsequently calculating the proportion of the lesions.

**Data analysis**

The data were first calculated in Excel 2021 (Beijing Jinshan Office Software Co., Ltd., Beijing, China). Subsequently, the data were statistically analyzed using GraphPad Prism 9.0 software (GraphPad Software Inc., La Jolla, CA, USA)


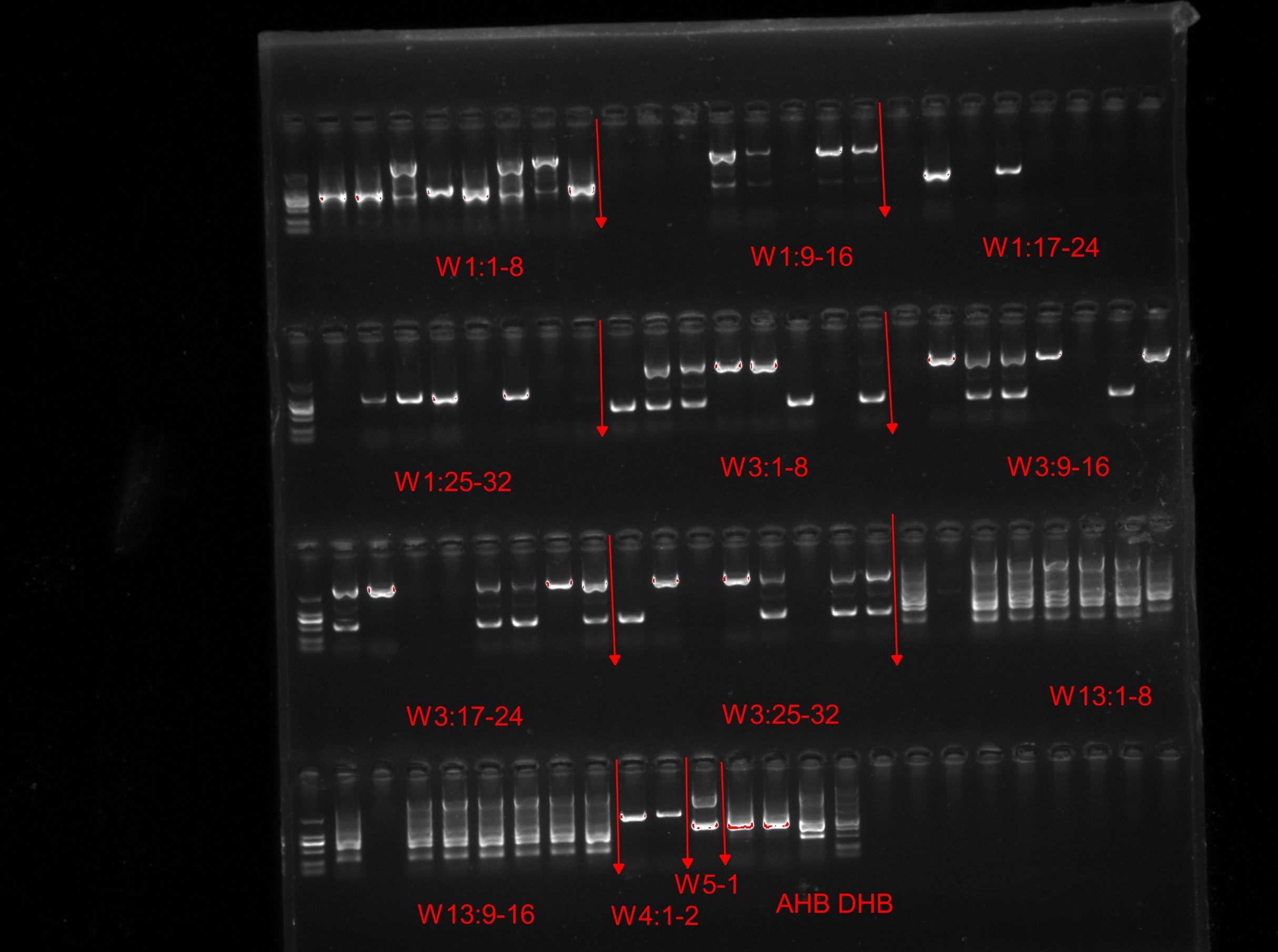

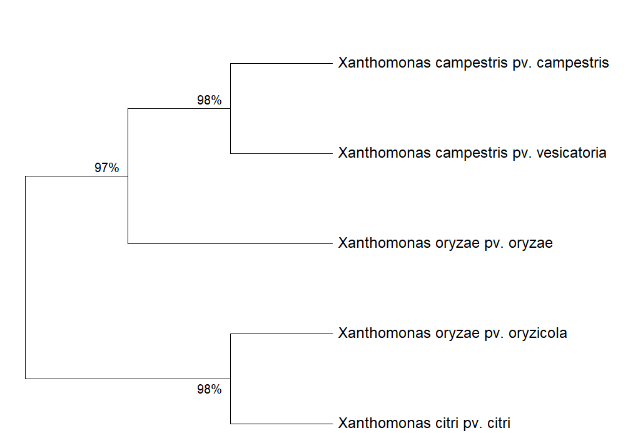


**Figure S1** (a) Validation of the AvrBs2 knockout mutant. The smaller band represents the successful knockout mutant, while the larger band corresponds to the wild-type strain. (b) Phylogenetic Analysis of AvrBs2 across Diverse *Xanthomonas* Strains.
